# Personalizing brain perfusion in traumatic brain injury

**DOI:** 10.62675/2965-2774.20260073

**Published:** 2026-03-16

**Authors:** Adrián Marcos-Morales, Geert Meyfroidt

**Affiliations:** 1 Hospital Universitario 12 de Octubre Servicio de Medicina Intensiva Madrid Spain Servicio de Medicina Intensiva, Hospital Universitario 12 de Octubre - Madrid, Spain.; 2 KU Leuven Department of Intensive Care and Hyperbaric Medicine Leuven Belgium Department of Intensive Care and Hyperbaric Medicine, KU Leuven - Leuven, Belgium.

## INTRODUCTION

Neuroprotective strategies in severe traumatic brain injury (TBI) patients focus primarily on optimizing the balance between cerebral oxygen and substrate delivery and the brain's metabolic demand.^(1)^ Achieving this balance is an immense challenge in the hours, days, and weeks following the initial injury, as many patients develop secondary insults such as hypoxia, hypotension, intracranial hypertension, or increased metabolic activity driven by neuro-inflammation, excitotoxicity, or seizures. Although personalized medicine guided by multimodal neuromonitoring – with early detection of secondary injury and individualized therapeutic targets – is widely advocated, clinicians still lack strong guidance from influential guidelines, which continue to rely on universal thresholds for all patients. This practical review summarizes key concepts of brain perfusion in TBI and outlines clinical management strategies, translating state-of-the-art methodologies into daily clinical practice.

## PRE-HOSPITAL AND EMERGENCY DEPARTMENT

According to the principles of ‘chain of trauma care’, preserving cerebral perfusion should be a priority in patients with suspected moderate or severe TBI, starting in the pre-hospital phase, and continuing throughout transport and emergency department care.^(2)^ In this setting, where intracranial pressure (ICP) monitoring is usually not yet available, targeting systolic blood pressure (SBP) ≥ 110mmHg or mean arterial blood pressure (MAP) > 80mmHg should ensure adequate cerebral perfusion, as mortality will increase with every 10-point drop in SBP < 120mmHg.^(3)^ Noninvasive point-of-care devices can help individualize care. Transcranial Doppler (TCD) can measure indirect parameters that depend on cerebral blood flow (CBF) and ICP. It is typically performed on the middle cerebral artery, where a high pulsatility index of ≥ 1.4 or low cerebral blood flow velocities (CBFV; mean < 30, diastolic < 20cm/s) can be suggestive of intracranial hypertension or inadequate brain perfusion. Quantitative pupillometry has a higher reliability than standard pupil evaluation and may assist in the early detection of intracranial hypertension.^(2)^

## IN THE NEURO-CRITICAL CARE UNIT

Intracranial pressure monitoring is essential for two reasons: it provides early warning of catastrophic mechanical injury or impending herniation, and it serves as a key indicator of cerebral hypoperfusion and secondary ischemic damage.^(4)^ In understanding ICP toxicity, the concept of ICP ‘dose’ or burden – capturing both the duration and intensity of elevated ICP episodes – is more informative than simple threshold crossing. Intracranial pressure values as low as 15mmHg can become harmful when sustained, and a higher ICP burden has consistently been associated with worse outcomes.^(5,6)^ Some patients also receive a brain tissue partial oxygen pressure (PbtO_2_) probe to monitor local brain tissue oxygenation. However, clinical trials (some still ongoing) have not yet demonstrated a clear mortality or functional outcomes benefit for this strategy, aside from a *post-hoc* signal in the OXY-TC trial in patients with initial intracranial hypertension.^(7)^ Low PbtO_2_ may reflect brain ischemia or brain hypoxia, and can be addressed by optimizing gas exchange, considering transfusion to maintain hemoglobin around 9g/dL, and targeting a cerebral perfusion pressure (CPP) around 70mmHg or above.^(8)^

Cerebral perfusion pressure is the difference between MAP and ICP. An often overlooked issue is the position of the blood pressure transducer: for accurate CPP calculation, it must be aligned with the level of the external auditory meatus or tragus,^(9)^ not with the traditional phlebostatic axis. In TBI patients with a head of bed routinely elevated 30 - 45°, using the phlebostatic axis will substantially overestimate CPP by up to 23mmHg, thereby masking episodes of low CPP.

Initial CPP management targets a value between 60 and 70mmHg.^(10)^ Similar to ICP burden, the cumulative ‘dose’ of low CPP is what drives harm, and CPP should never fall below 50mmHg.^(9)^ Cerebrovascular autoregulation (CA) is the endogenous mechanism that maintains CBF despite fluctuations in CPP. Cerebrovascular autoregulation impairment is common in severe TBI, reduces the brain's tolerance to these variations, and is associated with worse outcomes.^(11)^ In general, CA impairment increases the brain's vulnerability to both high-ICP insults and low-CPP insults, although the consequences of CA dysfunction at high CPP remain less well-defined.

There is no gold standard to measure CA in TBI patients at the bedside. Cerebrovascular autoregulation can be dynamically assessed by correlating spontaneous arterial blood pressure fluctuations with surrogate signals of CBF, such as ICP, PbtO_2_, TCD-derived CBFV, or near-infrared spectroscopy (NIRS)-based oxygenation. Indices like the pressure reactivity index (PRx), brain tissue oxygen reactivity index (ORx), mean flow index (Mx), and low frequency autoregulation index (LAx) quantify this pressure-flow relationship and indicate whether autoregulation is intact or impaired. Static assessment can be performed using a MAP challenge, raising or lowering MAP by ~10mmHg and observing the corresponding ICP, PbtO_2_, or CBFV response. Because no single method is definitive, bedside practice relies on a multimodal combination of these dynamic indices and MAP-challenge responses.^(9,10)^ In a given time window of at least 30 minutes, after gathering several PRx values, the CPP with the lowest PRx has been called the "optimal CPP", or CPPopt.^(11)^ Core and advanced tools for multimodal brain monitoring are further summarized in table 1.

**Table 1:** Core and advanced tools for multimodal brain monitoring

	Initial care (prehospital or emergency department)	Core tools after monitoring	Advanced tools after monitoring (research hospitals, case-by-case basis)
ABP (mmHg)	Avoid systolic ABP < 110 if severe TBI is suspected	Target MAP 80 - 100. Titrate to resuscitation endpoints (urine output, serum lactate, skin perfusion)	If hemodynamically stable, ABP management is subordinate to CPP
ICP (mmHg)	If high ICP is suspected (anisocoria, mydriasis), consider hyperventilation, hyperosmolar therapy, or sedation	Tier-based management if ICP > 22	Tier-based management, measure high ICP burden, and prevent ICP insults based on intensity and duration
CPP (mmHg)	-	Aim for default target 60 - 70mmHg. Avoid CPP below 50mmHg	Measure and adopt CPPopt, always within the security margin between 50 and 100
TCD	Potential trigger for ICP or CPP-directed therapy before CT and/or invasive monitoring available	Objectives CBFV: - Pulsatility index < 1.4 - Diastolic velocity of > 20cm/s - Mean velocity of > 30cm/s	Same objectives. Consider TCD-based methods for CA monitoring Screen for vasospasm
PbtO_2_ (mmHg)	-	Not always available Objective ≥ 20. Avoid < 15 for more than 30 minutes	Same objectives Could detect low CPP insults
Cerebrovascular autoregulation (CA)*	-	MAP challenge (increase MAP for 10mmHg up to 20 minutes) ✓ Choose a new higher CPP target if ICP lowers ✗ Abort the challenge if ICP rises	Continuous measurements with bedside integration software (PRx, LAx) Measure and adopt CPPopt If CA intact, low CPP could induce vasodilation and a rise in ICP

ABP - arterial blood pressure; TBI - traumatic brain injury; MAP - mean arterial pressure; CPP - cerebral perfusion pressure; ICP - intracranial pressure; CPPopt - optimal cerebral perfusion pressure; TCD - transcranial Doppler; CT - computerized tomography; CBFV - cerebral blood flow velocity; CA - cerebrovascular autoregulation; PbtO_2_ - brain tissue partial oxygen pressure; PRx - pressure reactivity index; Lax - low frequency autoregulation index.

* If intracranial pressure, cerebral perfusion pressure or brain tissue partial oxygen pressure are altered, their correction is a priority over obtaining more information on cerebrovascular autoregulation status.

**Figure 1:**
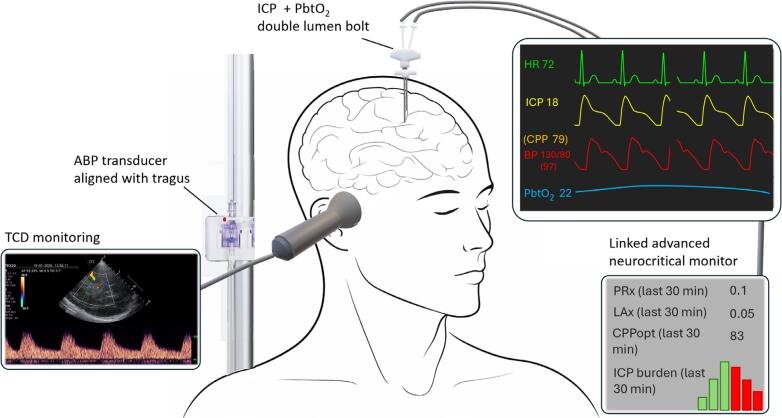
Personalizing brain perfusion in traumatic brain injury at the bedside.

The COGiTATE randomized controlled trial^(12)^ investigated the safety and feasibility of CPP optimization guided by continuous CA monitoring. The control group received standard CPP management targeting 60 - 70mmHg. In contrast, the intervention group received a real-time PRx-based CPPopt recommendation every 4 hours, which clinicians could adopt or reject. Figure 1 shows a similar setup. These recommendations were followed in 90% of cases and, in the intervention group, generally led to higher CPP values. The most frequently suggested CPP ranges were 60 - 70mmHg (49%), 70 - 80mmHg (31%), and 80 - 90mmHg (15%). The trial demonstrated that this PRx-guided protocol is both feasible and safe at the bedside.

ICP - intracranial pressure, PbtO_2_ - brain tissue partial oxygen pressure; ABP - arterial blood pressure; HR - heart rate; CPP - cerebral perfusion pressure; BP - blood pressure; TCD - transcranial doppler; TBI - traumatic brain injury; PRx - pressure reactivity index; Lax - low frequency autoregulation index; CPPopt - optimal cerebral perfusion pressure.

Further directions in cerebral perfusion research for TBI include direct CBF measurement techniques to validate CA and CPPopt-based therapies, as well as the implementation of machine learning models capable of predicting future episodes of elevated ICP.^(13)^ Functional ultrasound^(14)^ and optic non-invasive technologies^(15)^ are increasingly studied tools for CBF evaluation, in both animal models and humans.

## CONCLUSION

To bridge the gap between animal studies and clinical practice, new devices will need to be non-invasive (transcutaneous, software-based) or small enough to be introduced through the existing intracranial pressure burr hole. Functional ultrasound may enable direct assessment of cerebral blood flow, while diffuse optical monitoring techniques could yield non-invasive estimates of intracranial pressure and partial oxygen pressure in brain tissue. In the near future, a personalized protocol integrating optimal cerebral perfusion pressure, secondary insult prediction, and tailored tier-based management may offer a path towards improved patient outcomes.

## Data Availability

The contents are already available.
